# Impact of Fermentation and Pasteurization on the Physico-Chemical and Phytochemical Composition of *Opuntia ficus-indica* Juices

**DOI:** 10.3390/foods12112096

**Published:** 2023-05-23

**Authors:** Ricardo M. Ferreira, Adriana M. Costa, Carlos A. Pinto, Artur M. S. Silva, Jorge A. Saraiva, Susana M. Cardoso

**Affiliations:** LAQV-REQUIMTE, Department of Chemistry, Campus Universitário de Santiago, University of Aveiro, 3810-193 Aveiro, Portugal; ric.ferreira@ua.pt (R.M.F.); carlospinto@ua.pt (C.A.P.); jorgesaraiva@ua.pt (J.A.S.)

**Keywords:** *Opuntia ficus-indica*, prickly pear, fermentation, high pressure, temperature, phytochemical, antioxidant

## Abstract

*Opuntia ficus-indica* fruits are a source of valuable compounds, presenting a high nutritional value and several health benefits. However, due to its low shelf life and increased production, there are considerable post-harvest losses of this cactus fruit. So, ways need to be found to drain the increased production of this fruit that is being wasted. The chemical composition of prickly pear makes it an appealing substrate for fermentation. This study investigates the production of fermented beverages produced from *Opuntia ficus-indica* cv ‘Rossa’ and evaluates the effects of different fermentation times (18 and 42 h) and post-fermentation pasteurization by high-pressure (500 MPa for 10 min) and temperature (71.1 °C for 30 s) on the physico-chemical and biological characteristics of the produced beverages. According to the results, the beverage produced from 48 h of fermentation has an alcohol content value of 4.90 ± 0.08% (*v*/*v*) and a pH of 3.91 ± 0.03. These values contribute to an extended shelf life and improved organoleptic characteristics compared to the sample fermented for 18 h. Additionally, the longer fermentation resulted in 50% fewer total soluble solids, 90% less turbidity, and lower pH when compared to the sample fermented for 18 h. Moreover, overall, high-pressure processing demonstrates better retention of “fresh-like” characteristics, along with higher levels of phytochemical compounds and antioxidant capacity, similar to those observed in the juice for SO^•^- and NO^•^-scavenging abilities.

## 1. Introduction

*Opuntia ficus-indica* (L.) Mill., commonly known as prickly pear, is a species of cactus that is indigenous to Mexico and has been widely cultivated in various regions of the world, including Africa, Australia, and the Mediterranean basin [1]. This plant belongs to the Cactaceae family and is one of the most important genera, along with *Nopalea*, because of its diverse applications, such as the production of edible fruit, juices, jams, gels, or animal feed [2]. *Opuntia* and *Nopalea* are highly adaptable to harsh environmental conditions, including extended periods of drought, exposure to UV radiation, and low-quality soils [3]. 

Nowadays, *Opuntia* fruits and cladodes (i.e., stem pads responsible for performing photosynthesis) are considered functional foods, or healthy ingredients of new foods, since they provide interesting bioactive compounds, including fiber, hydrocolloids (mucilage), pigments (betalains and carotenoids), minerals (calcium and potassium), and vitamins with antioxidant properties, such as vitamin C, overall presenting a high nutritional value, as well as health benefits, namely for the prevention of several diseases [4,5]. The pulp is characterized by a high water content (84% to 90%) and reducing sugars like glucose and fructose (10% to 15%), 0.3% ash, and less than 1% protein [6]. The high soluble solids content makes it a very attractive medium for the growth of microorganisms [7], and its high pH implies its classification as a low-acidic food (pH > 4.5), requiring treatment to control the growth of pathogenic microorganisms [8]. Because of this, fruits and juices of prickly pear have a high deterioration tendency and a short harvest time of 3 to 4 weeks, which represents a problem for the commercialization of *Opuntia* fresh products [9]. 

Fermentation is a metabolic process that generates energy from sugar or other molecules in the absence of oxygen or an electron transport system. This process produces organic acids that reduce pH and increase the shelf life of products. While natural fermentation has been traditionally used to preserve foods, modern large-scale production utilizes well-characterized strains that ensure consistency and quality in the final product [10]. Fermented foods and beverages of both plant and animal origin have been produced and consumed worldwide for thousands of years. Alcoholic fermentation is commonly utilized for processing, stabilizing, and preserving sugar-rich substrates such as fruit or fruit and vegetable juices [11]. Through optimized fermentation processes, it becomes possible to enhance the concentration of specific bioactive compounds, typically including phenolic compounds, vitamin C, and vitamins from the B complex. This enrichment serves the interests of both the industry and consumers, as it contributes to the nutritional and health-promoting attributes of the fermented products [12]. In addition to fermentation, the food industry employs various heat treatments, such as pasteurization and sterilization, to extend the shelf life of foods [13] by reducing microbial load and deactivating spoilage enzymes like peroxidase (POD), polyphenol oxidase (PPO), and pectin methylesterase (PME). Thermal processing remains the most widely used preservation method, with typical pasteurization conditions for fruit juices involving the application of temperatures such as 65 °C for 30 min, 77 °C for 1 min, and 88 °C for 15 s [2,13,14]. However, thermal processing can negatively impact various quality properties of food products. To overcome the drawbacks of thermal processing, nonthermal technologies, including high-pressure processing (HPP), have been investigated as alternatives. HPP has emerged as a promising approach for processing food and beverages as discussed by Özlem Tokuşoğlu in his review [15]. Different combinations of pressure, time, and temperature can be used to ensure food safety and to increase the retention of phenolic compounds and antioxidant capacity during storage. Due to the promising results obtained by other authors for *Opuntia* fruits, as evidenced by previous studies, this technique has gained importance in the processing of this fruit and its products [16,17,18]. This technique involves applying high levels of pressure to a food product to achieve microbial inactivation while preserving its quality attributes. This is a potentially advantageous alternative to traditional thermal processing in the processing of *Opuntia* fermented beverages. However, further research is necessary to obtain a comprehensive understanding of the impact of post-fermentation pasteurization on the quality and shelf life of *Opuntia*-based products and to determine the optimal processing parameters.

Despite several authors having previously studied the nutritional and functional properties of the prickly pear fruit as an antioxidant-rich food source, the literature reporting the impact of fermentation on prickly pear juices is scarce. Karabagias et al. used juices from *Opuntia ficus-indica* Mill. of the wild cultivars, grown in the regions of Lakonia, Western and Eastern Messinia (Greece) to produce a bio-functional alcoholic beverage [19], and a few other authors used prickly pear to produce wine [20,21,22]. Moreover, to our knowledge, the application of post-fermentation pasteurization using HPP on fermented prickly pear beverages has not yet been addressed. Therefore, the use of HPP for the processing of *Opuntia* fermented beverages represents a novel application of this technology and may offer potential advantages over traditional thermal processing methods. The objective of this study was to develop a fermented beverage from prickly pear juice from the ‘Rossa’ cultivar using *Saccharomyces cerevisiae* and to evaluate the impact of subsequent thermal or high-pressure pasteurization on the product. In sum, it was aimed at providing a comprehensive understanding of the effects of pasteurization techniques on the sensory, physicochemical, and microbial attributes of the fermented beverage and at identifying the optimal pasteurization strategy for achieving microbial safety while preserving product quality.

## 2. Materials and Methods

### 2.1. Microorganism

The alcoholic fermentations were carried out with *Saccharomyces cerevisiae* DSMZ 70468 using a lyophilized culture previously purchased from DSMZ—a German collection of microorganisms and cell cultures. This strain was cultured on yeast malt agar plates, according to the manufacturer’s instructions, and then incubated at 30 °C for 48 h.

### 2.2. Inoculum Preparation

A culture was prepared by inoculating a single colony in 100 mL of sterile culture medium (yeast malt broth) containing 5 g/L animal tissue peptide digest (peptone), 3 g/L yeast extract, 3 g/L malt extract, and 10 g/L dextrose, with the pH adjusted to 4 using 1 M citric. The culture was then incubated at 30 °C with constant agitation at 150 rpm for a duration of 36 h. The inoculum was considered ready for use when the optical density of the culture medium reached 0.8, as measured at a wavelength of 600 nm [23].

### 2.3. Prickly Pear Juice Preparation 

The cv ‘Rossa’ of prickly pear (*Opuntia ficus-indica*) pulp, acquired from Agricultural Cooperative, Figo d’Idanha in a polyethylene bag-in-box container with 3 L of pulp, was conveyed in a refrigerated camera to the laboratory. Upon arrival, this was centrifuged using a centrifuge (Heraeus Biofuge Stratos, Thermo Electron Corporation, Waltham, MA, USA) at 21,000× *g* for 10 min. The supernatant (the juice) was collected, frozen, and stored at −20 °C. Prior to each experiment, each sample was thawed at room temperature. Moreover, after that, a portion of the juices underwent fermentation followed by thermal and HPP pasteurization (as described in Section 2.4 and Section 2.5, respectively). These processed samples, along with the non-pasteurized fermented beverage serving as the control group, were stored at a temperature of 4 °C throughout the analysis period.

### 2.4. Preparation of the Fermented Beverage

The prepared inoculum using *Saccharomyces cerevisiae*, which activated in YPD medium, was, subsequently, centrifuged and washed using distilled water to eliminate any residual culture medium. The resultant pellet of *S. cerevisiae* was suspended in 5 mL of prickly pear juice to serve as the inoculum for the alcoholic fermentation process. Alcoholic fermentation was carried out in 20 mL glass flasks, with 0.5 mL of the inoculum mixture added to 16.5 mL of prickly pear juice. The flasks were then incubated at 30 °C and 150 rpm for two different time periods: 18 h (F18) and 42 h (F42).

### 2.5. High-Pressure Processing (HPP) and Temperature (TP) Pasteurization

Two pasteurization methods, namely high-pressure processing (HPP) and thermal pasteurization (TP), were applied to the fermented beverages F18 and F42. The HPP treatments were performed using industrial-scale equipment with a 55 L capacity (model 55, Hiperbaric, Burgos, Spain) with water used as the pressurization fluid. Fermented beverages (17 mL) previously placed in sealed polyamide and polyethylene bags (3 × 6 cm) were subjected to a 500 MPa treatment for 10 min at room temperature. After processing, the HPP-pasteurized samples (F18-HPP and F42-HPP) were immediately cooled in an ice bath, stored at 4 °C, and subsequently analyzed. 

TP was performed by submerging the thermally sealed polyamide and polyethylene bags (3 × 6 cm) with 17 mL of fermented juice in a thermostatic bath (Selecta Frigiterm 6000382, Barcelona, Spain) at 71.1 °C. The required time to reach the desired temperature (70 s) was previously estimated using a K type thermocouple connected to a digital thermometer and once the desired temperature was reached, the beverages were held in a water bath for 30 s, followed rapid cooling on ice. The TP-pasteurized samples (F18-TP and F42-TP) were stored at 4 °C and subsequently analyzed.

### 2.6. Microbial Load of the Fermented Beverages

Prickly pear fermented juice samples that were subjected to the different treatments was analyzed for counts of microbial load. Aseptically, 1.0 mL of each sample was homogenized with 9.0 mL of Ringer’s solution. Furthermore, decimal dilutions were made with the same diluent, and duplicates of dilutions were plated on the appropriate media: total aerobic psychrophiles (TAP) counts were assessed in plate count agar (20 ± 1 °C for 120 ± 3 h) (ISO 4833:2003); *Enterobacteriaceae* (ENT) counts were enumerated in violet red bile dextrose agar (37 °C for 24 ± 1 h) (ISO8523:1991); yeast and mold (YM) counts were quantified on Rose-Bengal chloramphenicol agar (25 ± 1 °C for 120 ± 3 h) (ISO 7954:1987). Enumeration of microbial colonies was performed by selecting petri dishes with a range of 15–300 colony-forming units (CFU), and the outcomes were expressed as log_10_ CFU/mL. 

### 2.7. Physical-Chemical Properties of the Fermented Beverages

#### 2.7.1. pH and Titratable Acidity

The pH values of the fermented prickly pear beverages were determined at 20 °C using a pH 50-14 electrode (Crison Instruments, S.A., Barcelona, Spain) that underwent previous proper calibration. Titratable acidity (TA) was evaluated by titrating 10 mL of the diluted fermented beverages (1 mL of sample in 9 mL of distilled water) with a standardized 0.01 M NaOH solution until a pH of 8.1 was achieved. The outcomes were expressed as the concentration of citric acid in grams per liter of sample (g citric acid/L). An automatic titrator (Titromatic 1S, Crison Instruments, SA, Barcelona, Spain) was employed to carry out the titration process, and the procedure adhered to the equations outlined in reference [24]:

$$TA\left(g\;tartaric\;acid/L\right)=\frac{N\;NaOH\times mL\;NaOH\times 75}{mL\;of\;sample}$$

$$TA\left(g\;citric\;acid/L\right)=TA\left(g\;tartaric\;acid/L\right)\times 0.853$$

#### 2.7.2. Total Soluble Solids (°Brix), Browning, and Turbidity 

The determination of total soluble solids content was conducted by measuring the refractive index in terms of °Brix using an Atago Portable Refractometer (ATC-1E) at a temperature of 20 °C, and the results were expressed in °Brix. The degree of browning was quantified by subjecting the various samples to centrifugation at 9000× *g* and 4 °C for 20 min, followed by measuring the absorbance of the supernatant at 420 nm using a UV-Vis microplate spectrophotometer (Multiskan GO Microplate Spectrophotometer, Thermo Scientific, Thermo Fisher Scientific Inc., Waltham, MA, USA). Turbidity was gauged using the aforementioned UV-Vis microplate spectrophotometer by directly measuring the absorbance at 700 nm.

#### 2.7.3. Reducing Sugars

The quantification of reducing sugars present in the sample was carried out using a method previously described by Lopes et al. [25] through the 3,5-dinitrosalicylic acid (DNS) method. Specifically, 1.0 mL of the sample was mixed with 1.0 mL of DNS reagent and heated with boiling water for a duration of 5 min before cooling in an ice bath. Then, 10 mL of distilled water was added to dilute the mixture, and the absorbance of the resultant solution was measured at 540 nm using an automated plate reader (Biotek Instrument Inc., Winooski, VT, USA). Concentration values were determined using a calibration curve constructed with standard glucose solutions and expressed in mg/mL of the sample.

### 2.8. Alcohol Content 

To determine the alcohol content of the fermented samples, the method proposed by Papazian [26] was used. The specific gravity (SG) scale was used when measuring the °Brix. To obtain the alcohol percentage by weight, the specific gravity of the prickly pear juice was subtracted from the gravity of each fermented sample and multiplied by 105. Since alcohol is lighter than water, a measured volume of water is not equal in weight to an equal volume of alcohol. To convert the percent of alcohol by weight to the percent of alcohol by volume, the value obtained earlier was multiplied by 1.25.

### 2.9. Enzymatic Activity 

The enzymatic activities of peroxidase (POD) and polyphenol oxidase (PPO) were determined following the protocol established by Pinto et al. [27]. For the POD activity analysis, 10 μL of the sample aliquots were combined with 0.36 mM ABTS and 0.1 M sodium acetate buffer (pH 6.0), resulting in a final reaction volume of 1.9 mL, which was pre-incubated at 20 °C. Subsequently, the enzymatic reaction was initiated by the addition of 100 μL of 0.5 M hydrogen peroxide, and the formation of the ABTS cationic radical was continuously monitored at a wavelength of 414 nm using a Lambda 35 UV/Vis spectrometer (PerkinElmer Instruments Inc., Waltham, MA, USA) over a duration of 5 min.

To assess the activity of PPO, 0.6 mL aliquots of the fermented prickly pear beverage were combined with 2.4 mL of a substrate solution at 30 °C. The substrate solution contained 100 mM catechol in 100 mM citrate buffer (pH 5.4) and the absorbance of the reaction mixture was measured at 420 nm using a Lambda 35 UV/Vis spectrometer (PerkinElmer Instruments Inc., Waltham, MA, USA).

The pectin methylesterase (PME) activity was determined using a titration method as described by Ferreira et al. [18]. To perform the assay, 4 mL of fermented beverage was mixed with 2 mL of a 1% pectin solution in 250 mM NaCl [18]. The samples were titrated with 0.01 M NaOH while maintaining a constant pH of 7. NaOH consumption was recorded at 30 s intervals over a period of 15 min. The respective enzyme activity results were expressed as V (NaOH)/min/mL.

Enzymatic activities were obtained from the linear portion of the absorbance-time curves and expressed as ΔAbs/min/mL at 420 and 414, respectively, for PPO and POD, while for PME the ratio of NaOH/min/mL was used to determine the activity. 

### 2.10. Phytochemicals

#### 2.10.1. Total Phenolic Content

The total phenolic content of the samples was assessed using the Folin–Ciocalteu reagent, employing the adapted method from Silva et al. [28]. In summary, 15 µL of the diluted sample was mixed with 15 µL of Folin–Ciocalteu reagent and 60 µL of distilled water for 5 min. Following this, 150 µL of sodium carbonate solution (7%) was added to the reaction mixture, and the resulting solution was incubated at room temperature for 1 h. The absorbance readings were then recorded at 750 nm using an automated plate reader (Biotek Instrument Inc., Winooski, VT, USA). Gallic acid was used as a reference standard, and the results were expressed in terms of milligrams of gallic acid equivalent (GAE) per milliliter of sample (mg GAE/mL).

#### 2.10.2. Total Flavonoid Content

The total flavonoid content in the fermented samples was determined by the colorimetric method (aluminum trichloride method), as described by Ferreira et al. [18]. Briefly, 150 µL of fermented juice sample was added to 150 µL of 2% aluminum trichloride solution (AlCl_3_-2H_2_O). The mixture was stirred and allowed to stand for 1 h reaction time. The absorbance was measured at 420 nm with a UV-Vis spectrophotometer (Biotek Instrument Inc., Winooski, VT, USA). The concentration of flavonoids was determined by comparing the absorbance values of the sample with the absorbance values of quercetin used as a standard. The results were expressed as quercetin equivalents (QEq. mg/mL sample) ± SD (standard deviation). 

#### 2.10.3. Total Betalain Content

The method described by Stintzing et al. was used to quantify the total betalain content of prickly pear fermented juices [29]. The samples were placed directly into the microplates for reading the respective absorbances. The betalain content (BC) was calculated as described previously: BC [mg/L]) [(A × DF × MW × 1000/ε × l)], where A is the absorbance value corrected for absorbance at 600 nm, DF is the dilution factor, and l is the path length of the cuvette in centimeters. For the quantification of betacyanins and betaxanthin, the molecular weights (MW) and molar extinction coefficients (ε) of betanin (MW = 550 g/mol; ε = 60,000 L/(mol cm) in H_2_O; λ = 538 nm) and indicaxanthin (MW = 308 g/mol; ε = 48,000 L/(mol cm) in H_2_O; λ = 480 nm) were applied. All measurements were performed in duplicate using an automated UV-Vis plate reader (Biotek Instrument Inc, Winooski, VT, USA).

#### 2.10.4. Identification of Phenolic Compounds by Ultra-High-Performance Liquid Chromatography with Photodiode Array Detector and Mass Spectrometry (UHPLC-DAD-ESI-MS^n^)

The UHPLC-DAD-ESI/MS studies were performed with the help of an Ultimate 3000 system (Dionex Co., San Jose, CA, USA), which included an auto-sampler, a quaternary pump, an Ultimate 3000 diode array detector (Dionex Co., San Jose, CA, USA), and an automated thermostatic column compartment. This system was linked to an ion trap MS using an electrospray ionization (ESI) source (Thermo LTQ XL MS, Thermo Scientific, San Jose, CA, USA). Thermo Scientific’s Thermo Xcalibur Qual Browser data system (San Jose, CA, USA) was used for control and data collecting. To make the analysis easier, high-purity nitrogen gas (>99%) was used at 520 kPa (75 psi). The device was set to run in negative-ion mode, with a scanning range covering the mass range from m/z 100 to 2000. The ESI needle voltage was set to 4.80 kV, and the ESI capillary temperature was maintained at 275 °C. For efficient separation, a Hypersil GOLD C18 column (100 mm length, 2.1 mm i.d., 1.9 m particle diameter, end capped from Thermo Scientific, Waltham, MA, USA) was used, and the column temperature was maintained at 30 °C. With a flow rate of 0.200 mL/min, the mobile phase was a gradient elution of 0.1% (*v*/*v*) formic acid in water (solvent A) and 30% (*v*/*v*) methanol in acetonitrile (solvent B). The gradient program began with 5% solvent B and grew to 40% after 14 min, followed by a quick increase to 100% after 16 min. This state was maintained for 2 min before reverting to the starting conditions after 20 min. During the study, UV-Vis spectral data for all peaks were collected in the 200–700 nm range, and chromatographic profiles were observed at 280 nm. Compound identification was accomplished by comparing retention durations, absorption spectra, and MS data to recognized standards and the relevant literature [18,30].

### 2.11. Antioxidant Activity of the Fermented Beverages

The total antioxidant activity of the fermented juice samples was measured using an adaptation of the ABTS^•+^ [31], DPPH^•^, superoxide radical (SO^•^), and nitric oxide radial (NO^•^) [32] decolorization assays, as well as the capacity to reduce iron (III)-reducing power [33]. The results were expressed as gallic acid equivalents per milliliter of sample (GAE/mL) for the ABTS^•+^, DPPH^•^, SO^•^, and NO^•^ and milligrams of BHA equivalents (BHAE) per milliliter (mL) of sample for the iron-reducing activity power assay. Measurements were conducted using an automated plate reader (Biotek Instrument Inc., Winooski, VT, USA) at 734 nm, 517 nm, 560 nm and 562 nm, respectively, and appropriate dilutions of standards and samples were prepared for accurate analysis.

### 2.12. Statistical Analysis 

The results of the various analyses are presented as mean ± standard deviation of at least three independent assays performed in duplicate. All the above experimental data were subjected to statistical analysis using GraphPad software to evaluate significant differences between samples. The analysis was performed by ANOVA test with a significance level of 0.05.

## 3. Results

### 3.1. Microbial Load 

**Figure 1:**
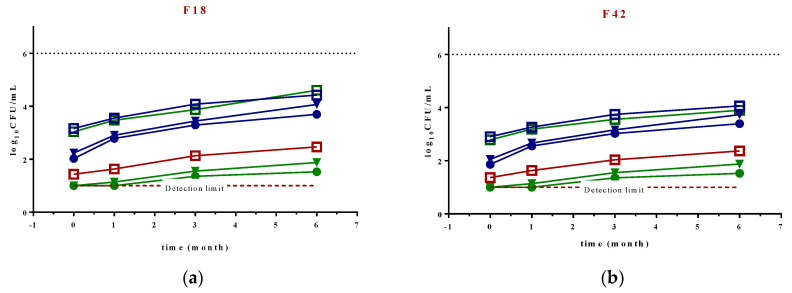
Evaluation of the total aerobic psychrophiles (blue), *Enterobacteriaceae* (red), and yeast and mold (green) counts (expressed in Log_10_ CFU/mL) in a fermented beverage of *O. ficus-indica* cultivar ‘Rossa’ (red pulp) (**a**) fermented during 18 h (F18) and (**b**) fermented during 42 h (F42) (non-pasteurized—□, pasteurized with high-pressure—●, pasteurized with temperature—▼; unsuitable for consumption (6.0 log)—••••, and ---- below the detection limit). Samples of *Enterobacteriaceae* for both HPP and thermal pasteurization are not represented in the graphics, as values were below the detection limit (1.0 log). Results are expressed as the average ± SD (n = 3).

Microbial load refers to the quantity and composition of bacteria present in an object or organism, which is crucial for assessing food quality and safety. In the present study, microbiological quality of F18 and F42 was followed by monitoring the TAP, *Enterobacteriaceae*, and yeast and mold counts (Figure 1a,b, respectively) during storage at 4 °C for 6 months. In this period, none of the samples achieved the threshold of spoilage of ≥ 6.00 Log_10_ CFU/mL for total microbial counts [27]. The initial populations of viable microorganisms for both fermented beverages were roughly 2–3 Log_10_ CFU/mL for TAP, 1.5 Log_10_ CFU/mL for Enterobacteriaceae, and 3 Log_10_ CFU/mL for yeast and mold. Comparable results were found in fresh fruit from *O. ficus-indica Mill*, cv. ‘Gialla’ from Italy (storage under modified environment) and ultrasound-treated green prickly pear juice from Mexico, in which total aerobic psychrophilic counts were in the range of 3.20–4.60 Log_10_ CFU/mL [34,35]. Notably, the initial microbial load of *Enterobacteriaceae* was similar to those reported in the literature for *Opuntia* juices (approximately 1.61 Log_10_ CFU/mL), while yeast and mold counts were around two times higher [36,37,38] because of the presence of *Saccharomyces cerevisiae* used in the fermentation process. *Saccharomyces* spp. are widely recognized as a prominent probiotic, meeting the World Health Organization’s definition of a “live microorganism capable of conferring multiple health benefits on the host when administered in adequate amounts”. The presence of this microorganism offers several advantages, including heightened enzymatic activity, enhanced intestinal barrier functionality, production of antibacterial or bactericidal compounds, modulation of host immune responses, and influence on intestinal carcinogenesis [12].

In the 6-month period studied, TAP levels increased around 1 Log_10_ CFU/mL for both the unpasteurized and pasteurized samples. In turn, the levels of *Enterobacteriaceae* in the treated samples were below the detection limit, which is due to the high efficiency of both pasteurization methods in the inactivation and reduction in *Enterobacteriaceae* family microorganisms [39]. Additionally, the high concentration of betalains present in the prickly pear beverage examined in this study may have contributed to the low concentration of these microorganisms during storage, as betalains have the ability to chelate essential inner cations such as Ca^2+^, Fe^2+^, and Mg^2+^ in bacteria, leading to cell death [40].

As expected for untreated fermented beverages, yeast and mold counts were higher compared to those of pasteurized samples, since both temperature and pressure can effectively inactivate yeast and mold [41]. Although some microbial growth was observed in samples that underwent thermal treatment and high-pressure processing over time at 4 °C, the initial reduction in microbial load achieved by both treatments resulted in significant differences after the 6-month study period. These findings are consistent with previous reports, indicating that a slight increase in the total microbiological count of beverages such as cider, beer, fruit wines, refined rice wine, and yakju may occur during storage [42,43]. 

### 3.2. Physical-Chemical Analysis 

#### pH and Titratable Acidity

In the context of fermentation and alcoholic beverages, pH is also indicative of the concentration of organic acids present in the solution, which can be quantified as titratable acidity. In this study, the pH and titratable acidity (Table 1) of the prickly pear juice prior to fermentation were 5.84 ± 0.14 and 0.049 ± 0.002 g_citric acid_/L, respectively, which are consistent with previously reported values for prickly pear juices derived from different cultivars [5,18,44,45]. The onset of alcoholic fermentation resulted in a decline in pH, primarily because of the production of organic acids by yeast, as a result of their physiological activity [46,47]. The pH values observed in this study align with the microbial reaction to acid stress, wherein the cells strive to maintain the intracellular pH above a critical threshold level. This critical threshold level, as reported by Lund et al., ranges from 2.9 to 4. Therefore, the obtained pH values are indicative of the microbial mechanism of acid tolerance and support the notion that cells aim to regulate their internal pH to counteract acid stress [48]. Moreover, the pH values used in this study to produce prickly pear beverage were in line with those employed by other researchers for the production of prickly pear wine [20,21]. As expected, a prolonged fermentation period of 42 h resulted in a higher increase in the titratable acidity of the fermented beverage. This rise in titratable acidity is indicative of a higher total acidic content relative to the 18 h fermentation period and is accompanied by a reduction in pH, which is also observed with prolonged fermentation time.

The application of HPP and TP pasteurization caused a decrease in titratable acid by approximately 40% in F18 and F42. Related to this, when evaluating the effects of HPP treatment (100–500 MPa for 10–60 min) on the chemical properties of young red wine, Sun and coworkers found that it significantly reduced the contents of tartaric acid [49]. In another study, Boynton et al. also obtained reduced titratable acidity of mangos after HPP treatment (600 MPa for 1 min), accompanied by a reduction in the pH [50]. These findings suggest that pressure can affect the content of organic acids and may account for the lowering of titratable acidity and pH values observed in the HPP-processed samples, compared to the temperature-processed and unprocessed fermented samples.

### 3.3. Total Soluble Solids (TSS) (°Brix), Browning, and Turbidity

The results of total soluble solids (TSS), browning degree, and turbidity of the juice and of fermented beverages of *O. ficus-indica* cv ‘Rossa’ are summarized in Table 2. In the context of fermentation, TSS plays a significant role as it provides the fermenting microorganisms with a source of nutrients, particularly sugars, which serve as substrates for their metabolic activities. In general, TSS in fruits and vegetables represent the content of sugars, mainly glucose, fructose and sucrose, organic acids, and other minor constituents. The initial °Brix of the prickly pear cv ‘Rossa’ juices was 13.10 ± 0.21, which is similar to that reported in the literature in a previous study for juices from this fruit [5,8,18,29,44,45]. During fermentation, the cultures of *S. cerevisiae* use the free sugars from the juice to produce energy, thus reducing the °Brix. So, during the fermentation of prickly juices, a reduction in °Brix was expected. Moreover, the increase in fermentation time from F18 to F42 should also be accompanied by a slight variation in the total soluble solids present in the final product. The results of the F18 and F42 samples are similar to those found for artisanal beers, 6.66–6.75 °Brix, and red apple ciders, 4.1–4.3 °Brix, respectively, which possess similar initial values of TSS but longer fermentation times [51,52]. 

The degree of browning in commercial beverages is an important quality parameter, as high levels of brown pigments can reduce the visual appeal of the final product, leading to consumer rejection [53]. The browning process is complex and is influenced by several factors, including the activity of polyphenol oxidase, pH, and polyphenol concentration. Companies therefore seek methods to mitigate the negative impact of browning. Fermentation is known to be associated with a reduction in the browning degree, as reported by Li et al. for fermented pear juice when compared with non-fermented samples [54].

In this study, the browning index of the prickly pear juice sample was 1.309 ± 0.082, which is similar to previous reports for the same juice [18], albeit slightly higher than values reported in the literature for prickly pear juices from Algeria [55], possibly because of the effect of enzymatic browning caused by the storage of the juice at −20 °C prior to use [56]. Browning tended to decrease with the fermentation process, as well as with pasteurization. Notably, between the two pasteurization methods, TP was more effective in reducing the browning degree. This may be due to the a better ability to inactivativate some oxidative enzymes (PPO, POD, and PME) [57]. This point will be further detailed in Section 3.5. 

Turbidity in a liquid is attributable to the presence of suspended particles, such as cells, proteins, or other insoluble substances. In the context of fermentation, turbidity serves as an indicator of the abundance of microbial cells, including yeast, as well as other particulate matter [58]. Throughout the fermentation process, the turbidity of the juice increased, resulting in higher turbidity values in the final fermented beverages. Values for F18 and F18-TP were close to 2, while those of F18-HPP were about half. This may be because the thermal process used did not totally destroy the *S. cerevisiae*: this remaining in suspension and thus affecting its turbidity. On the other hand, F42 samples presented lower values than F18, probably because of the prolonged fermentation process, and there was a drastic decrease in available sugars, while the high amounts of alcohol concentration present in the fermented sample may have caused the accumulation of harmful compounds for the *S. cerevisiae*, providing its precipitation and facilitating the separation [9,59]. Moreover, the prolonged fermentation allowed for greater fragmentation of sample components and the reduction in the size of suspended particles, leading to a lower content of total soluble solids in these samples and a reduced turbidity. F42-HPP turbidity was even lower than in the other two samples. HPP is well-known for its effects in reducing the turbidity of other fruits and vegetables [58]. 

### 3.4. Reducing Sugars and Alcohol Content 

A reducing sugar is characterized by the presence of free aldehyde or ketone functional groups within its molecular structure. These functional groups can undergo oxidation-reduction reactions, thereby facilitating the conversion of reducing sugars into other compounds during chemical or biological processes such as fermentation [18]. The concentration of fermentable reducing sugars in the samples was assessed using the DNS method, with a value of 158.87 ± 18.07 mg/mL registered for the prickly pear juice (Table 2). This value is slightly higher than those reported in the literature for prickly pear juice, a fact that is justified by several factors, such as cultivation conditions and degree of fruit maturation. For instance, in our prior research, we found values of 90 mg/mL for prickly pear juice cv ‘Rossa’ from Portugal from a different harvest [18]. Meanwhile, levels of reducing sugars in juices obtained from Mexican species of *Opuntia* were in the range of 50 to 140 mg/mL [60]. As expected, the concentration of fermentable sugars decreased over the course of fermentation, as *S. cerevisiae* converts glucose and fructose (which are the two predominant reducing sugars in prickly pear juice) into ethanol. Values of reducing sugar in F18 were close to 60 mg/mL, indicating that the fermentative process was still ongoing and that *S. cerevisiae* had not utilized all available substrate to carry out alcoholic fermentation. The reducing sugar concentrations in F18-HPP and F18-TP were lower than those observed in the untreated samples, as pasteurization methods can cause degradation of reducing sugars present in the medium [18], with fructose being particularly sensitive to such treatments [61]. The values obtained in samples fermented for 42 h were about 1.5 to 2 times lower than those found in ciders, where reducing sugar levels have been reported to range from 4.73 ± 0.058 to 5.63 ± 0.058 mg/mL [62].

As reported, prolonging the fermentation time leads to a higher consumption of the available reducing sugars by *S. cerevisiae*, resulting in an increased conversion of these sugars into ethanol. This was demonstrated by the higher alcohol percentage in the latter samples (F42). Several studies have suggested that yeasts utilize dissolved solids as a substrate for growth and alcohol production [52], which supports our findings as TSS is related to the amount of alcohol that the yeast can produce from an initial juice sample. For every two °Brix, a typical yeast strain can potentially produce about 1% alcohol plus one atmosphere (a standard unit of measurement for bottled beverages) of dissolved CO_2_ [63], which validates the results obtained for the percentage of ethanol since the approximately 5% (*v*/*v*) was obtained for a 9 °Brix variation range. Retamal et al. stated that the ethanol production capacity of *S. cerevisiae* using fresh fruit is 5.45 mL/100 mL [64], which is only slightly higher than the 4.9 mL/100 mL obtained for F42. F42 had an alcohol content similar to that reported in the literature for ciders (5.9–6.2%) [52], beers (4.9% v) [65], colonche (4–6%) [5], a beverage fermented with a mixture of prickly pear juice and grape juice that showed values of 5.90 and 9.60%, and a six-day fermentation of prickly pear juice that yielded an alcohol content of 9 ± 0.31% (*v*/*v*) [21].

### 3.5. Enzymatic Activity 

Fruits and vegetables are known to contain oxidative enzymes such as PPO, peroxidase POD, and PME that play a significant role in the oxidation of various compounds. Among these, PPO is considered a spoilage enzyme that can lead to a reduction in the nutritional quality of food and the development of unfavorable flavor characteristics [52]. In addition, this enzyme is also associated with the formation of brown pigments in the presence of oxygen because it catalyzes the oxidation of polyphenols into orthoquinones, which polymerize rapidly, either with themselves or with amino acids or proteins, forming these pigments [53]. The comparison of the degree of browning of each sample (Section 3.3) with those of Table 3 allow us to conclude that, in general, there is a similar tendency in these parameters. Samples with less enzymatic activity presented lower browning indexes, as could be observed for F42. Prickly pear juice, which was not subjected to further processing, had, as expected, the highest value of this enzyme, 0.679 ± 0.026 ∆Abs_420_ nm/min/mL. It is noteworthy that the PPO activity registered for F18 was superior to that in F42, meaning that fermentation caused the reduction in its activity, as mentioned by some authors [66]. Moreover, as expected, PPO activity was also reduced by the pasteurization procedure, since both HPP and TP are well-known to inhibit PPO [57,67]. For HPP and TP fermented beverages, PPO activity was decreased by approximately 48% and 64%, compared to the respective non-pasteurized samples. 

PME acts on pectin and produces the different structural and functional properties responsible for phase separation and cloud loss in fruit juice manufacturing [68], which affects the overall quality of the final product. POD catalyzes the oxidation of many organic compounds by hydrogen peroxides such as amines, phenols, and hydroquinone, inferring the biological properties of plants and plant products. As also observed in Table 3, variations in the activity of PME and POD as a function of the fermentation time and pasteurization showed a similar trend to that of PPO. Fermentation reduced the activity of these enzymes, an effect that was more prominent with the increment of the fermentation time as reported by Cloughley, 1980, for the fermentation of black tea [69]. Moreover, the combination of a longer fermentation period with pasteurization led to the inhibition of PME and POD, once the first was not detected and the second had residual values. Similar results were obtained for prickly pear and orange juices after pasteurization [18,59]. The higher value detected for PME in F18 samples after HPP pasteurization may be justified by the persistence of a fraction of this enzyme that is resistant to high-pressure and which corresponds to the heat-stable fraction of PME, as stated by Timmermans et al. [59].

### 3.6. Phytochemicals 

Phytochemicals are bioactive compounds synthesized by plants. These compounds are abundantly present in various plant-based sources such as fruits, vegetables, grains, legumes, and other botanical organisms. They contribute to the diverse chemical composition of plants and possess potential health-promoting properties. The effects of fermentation time and posterior thermal and high-pressure pasteurization on the total phenolic compounds (TPC), total flavonoid compounds (TFC), and betalain content of *O. ficus-indica* cv ‘Rossa’ juices and beverages are summarized in Table 4. 

In general, the total phenolic content presented a slight tendency to decrease with increased fermentation time, similar to what was reported by Varo et al. for the winemaking process of *Vaccinium corymbosum* wine obtained by maceration [70]. Both pasteurization methods employed in this study resulted in a reduction in TPC compared to the non-pasteurized samples. This decline in TPC following pasteurization aligns with the observations made by Błaszczak et al. for aronia juices, where the application of various pressure levels led to significant decreases in total phenolic compounds [71]. The TPC of the juice was found to be 0.637 ± 0.133 mg_GAE_/mL, also in line with the literature reported values for Sicilian *Opuntia ficus-indica* (L.) Mill [72]. Moreover, Es-Sbata et al. reported similar variations in the production of prickly pear wine and vinegar from yellow-orange wild cultivars grown in the Marrakesh–Safi–Morocco region [73]. Regarding other fermented beverages, the literature values for traditional beers are in the range 0.321–0.446 mg_GAE_/L, and those of fruit beers in the range 0.399–0.767 mg_GAE_/L [74]. These values suggest that the total phenolic content of both types of beer fall within the same range of the ones obtained in this work. Moreover, the TPC measured for samples with similar fermentation times were similar, thus suggesting that the pasteurization technique may not significantly influence the total phenolic content of these beverages.

**Figure 2:**
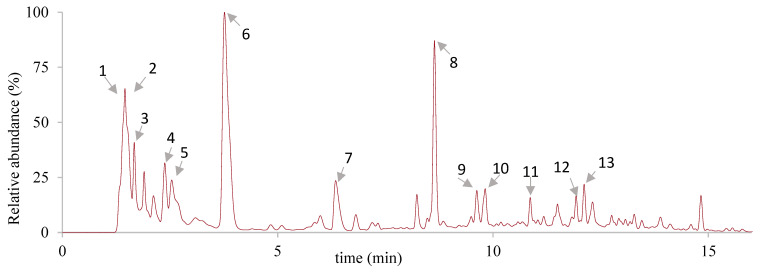
Chromatogram of *Opuntia ficus-indica* cv ‘Rossa’ juice fermented for 42 h after high-pressure pasteurization, at 280 nm, by UHPLC-DAD-ESI-MS^n^. Peak numbers correspond to those represented in Table 5. The chromatograms of the remaining samples present high similarity to this one and are not shown.

In addition, all the fermented beverages showed similar chromatographic peaks at 280 nm only with slight intensity variations (not shown), indicating that the phenolic constituents remained relatively unchanged, albeit in lower levels. This observation is consistent with a review by Giraldo-Silva et al., which discussed the phytochemical and pharmacological activities of different *O. ficus-indica* cultivars and reported similar phenolic compositions among studies conducted with different cultivars [75]. Moreover, in a previous work with juices from the same cultivars, the same compounds were also identified [18]. The representative chromatogram of these beverages is shown in Figure 2, and the main detected compounds are differentiated in Table 5. Notably, the most prominent peaks in the chromatogram of these samples corresponded to piscidic acid and eucomic acid, which are typically found in the fruit, as previously reported for *O. ficus-indica* juices [18,76]. In addition to these, the chromatograms showed the presence of distinct types of compounds, including the organic acids, quinic acid, malic acid, and citric acid, caffeic acid (cinnamic acid), and several isorhamnetin derivatives (flavon-3-ol).

### 3.7. Total Betalain Content

Betalains, well-known for exhibiting strong antioxidant properties, are derivates of two major compounds, betacyanin and betaxanthin, and their combination is responsible for the color exhibited by the fruit. However, they are susceptible to variations in pH, oxygen, metal ions, temperature, water activity, light exposure, and enzyme activities. During technological processing, the samples are susceptible to those alterations, which can accelerate their decomposition and influence their content [18,77]. 

As shown in Table 4 and as expected because of the sensibility of betalains to changes in oxygen levels, temperature, and light exposure, the concentration of total betalains decreased slightly with fermentation time [77]. Moreover, the application of thermal pasteurization accelerated the degradation of these compounds when compared to non-pasteurized samples and with HPP treatment. Betalains were reported to be preserved as a result of the acidity on the medium, it having been mentioned that they are relatively stable in a pH range between 3 and 7, which can explain the only slight variation between F18 and F42 [78]. 

### 3.8. Antioxidant Activity 

Antioxidants refer to synthetic or naturally occurring substances that have the potential to prevent or delay certain forms of cellular damage. Therefore, the consumption of fruits with a high content or capacity of antioxidants offers a multitude of health benefits. In this work, the antioxidant activity of juices and fermented beverages was screened by means of ABTS^•+^-, DPPH^•^-, NO^•^- and SO^•^-scavenging ability, and iron (III)-reduction capacity, using gallic acid as the reference standard. The overall gathered results demonstrated that an increase in fermentation time and the submission of the different juices/fermented beverages to pasteurization methods caused significant changes in the capacity to scavenge the radicals DPPH^•^, ABTS^•+^, SO^•^, and NO^•^ and the capacity of the fermented beverages to reduce iron (III) (Table 6). A small but general tendency toward a decrease in the antioxidant capacity could be observed over fermentation time. It is possible that this decrease is associated with the lowering in phytochemicals levels occurring with fermentation, as observed in Section 3.6 and as reported by Sawicki and Wiczkowski [79]. On the other hand, fermentation of 18 h led to an initial increase in the capacity to scavenge NO^•^, a fact that may be related with the anti-inflammatory effect of *Saccharomyces* [80], as previously reported for the fermentation of ginseng marc by Eom et al. [81]. For iron-reducing power, it was observed that the samples that underwent thermal processing exhibited the most significant difference, while the fermentation time and pasteurization by high-pressure processing had a negligible effect. Notably, F42 showed an increase in iron-reducing activity. The findings of this study were consistent with those of a previous investigation by Małgorzata Tabaszewska et al., who reported that lacto-fermented white asparagus exhibited an increased ferric-reducing capacity because of the favorable impact of fermentation [82]. Moreover, it can be observed that the application of the pasteurization methods for both fermentation times led to the increase in antioxidant capacity when compared with the results of the non-pasteurized sample. These findings align with the results reported by Błaszczak et al. in their study on *Actinidia arguta* fruits, specifically regarding ABTS^•+^ and iron-reducing assays [83].

## 4. Conclusions

After the two different fermentations, it was concluded that the 42 h fermented sample closely resembled cider, exhibiting an alcohol content of 4.90 ± 0.08% (*v*/*v*), pH of 3.91 ± 0.03, and low enzymatic activity, leading to a longer shelf life and improved physicochemical characteristics. Longer fermentation times showed increased physicochemical characteristics, indicating potential enhancement of the final product’s quality. Despite a small reduction in biological properties, the presence of similar compounds such as malic acid, piscidic acid, eucomic acid, and isorhamnetin derivatives was detected, suggesting the retention of important bioactive compounds despite the extended fermentation period. These changes in phytochemical and antioxidant capacity did not necessarily have negative impacts on the biological properties. Post-fermentation pasteurization preserved higher concentrations of phytochemicals and antioxidants, with lower values of TSS, turbidity, and enzymatic activity, resulting in improved overall outcomes compared to non-pasteurized samples. Among the two pasteurization techniques tested, high-pressure processing demonstrated the best preservation of these desirable characteristics. Considering the beneficial properties of prickly pear fruit combined with the appealing sensory attributes of the fermented beverage produced, there is potential for this product to become a functional beverage accepted by the average consumer. Moving forward, future perspectives may involve exploring different conditions to optimize the product’s physicochemical and biological properties. Additionally, conducting sensory evaluations and consumer acceptance studies could provide valuable insights into marketability and potential product variations.

## Figures and Tables

**Table 1:** pH and titratable acidity of *Opuntia ficus-indica* cv ‘Rossa’ juice and fermented beverages.

Sample	pH	Titratable Acidity (g_citric acid_/L)
Juice	5.84 ± 0.14 ^a^	0.049 ± 0.002 ^a^
F18	4.15 ± 0.04 ^b^	0.393 ± 0.010 ^b^
F18-HPP	4.11 ± 0.03 ^b^	0.178 ± 0.004 ^c^
F18-TP	4.31 ± 0.02 ^c^	0.222 ± 0.008 ^d^
F42	3.91 ± 0.03 ^d^	0.494 ± 0.005 ^e^
F42-HPP	3.84 ± 0.02 ^d^	0.283 ± 0.007 ^f^
F42-TP	4.10 ± 0.03 ^b^	0.328 ± 0.020 ^g^

F18 and F42- beverages obtained by fermentation for 18 h and 42 h, respectively, without pasteurization procedure; F18-HPP and F42-HPP- beverages obtained by fermentation for 18 h and 42 h, respectively, and post-fermentation pasteurization by high-pressure; F18-TP and F42-TP- beverages obtained by fermentation for 18 h and 42 h, respectively, and post-fermentation pasteurization by thermal pasteurization. Data marked with different letters are significantly different (ANOVA test; *p* ≤ 0.01) between treatments (in column) of the analyzed samples. Results are expressed as the mean ± SD (n = 3).

**Table 2:** Physico-chemical characteristics of *Opuntia ficus-indica* cv ‘Rossa’ juice and fermented beverages.

Sample	Total Soluble Solids (°Brix)	Browning	Turbidity	Reducing Sugars mg_sugar_/mL	Alcohol Content % (*v*/*v*)
Juice	13.10 ± 0.21 ^a^	1.309 ± 0.082 ^a^	0.432 ± 0.004 ^a^	158.87 ± 18.07 ^a^	-
F18	8.60 ± 0.00 ^b^	0.816 ± 0.025 ^b^	1.724 ± 0.034 ^b^	62.34 ± 5.13 ^b^	2.494 ± 0.000 ^a^
F18-HPP	8.60 ± 0.00 ^b^	0.837 ± 0.011 ^b^	0.970 ± 0.001 ^c^	49.56 ± 2.55 ^b^	2.494 ± 0.000 ^a^
F18-TP	7.67 ± 0.12 ^c^	0.768 ± 0.009 ^b^	1.969 ± 0.069 ^d^	39.88 ± 1.62 ^b^	2.975 ± 0.076 ^b^
F42	3.93 ± 0.12 ^d^	0.936 ± 0.004 ^c^	0.518 ± 0.025 ^ea^	2.123 ± 0.536 ^c^	4.900 ± 0.076 ^c^
F42-HPP	4.13 ± 0.06 ^d^	0.794 ± 0.005 ^db^	0.076 ± 0.006 ^f^	2.139 ± 0.676 ^c^	4.856 ± 0.000 ^c^
F42-TP	4.53 ± 0.12 ^e^	0.704 ± 0.001 ^eb^	0.160 ± 0.011 ^f^	3.120 ± 1.265 ^c^	4.637 ± 0.076 ^d^

F18 and F42- beverages obtained by fermentation for 18 h and 42 h, respectively, without pasteurization procedure; F18-HPP and F42-HPP- beverages obtained by fermentation for 18 h and 42 h, respectively, and post-fermentation pasteurization by high-pressure; F18-TP and F42-TP- beverages obtained by fermentation for 18 h and 42 h, respectively, and post-fermentation pasteurization by thermal pasteurization. Data marked with different letters are significantly different (ANOVA test; *p* ≤ 0.01) between treatments (in column) of the analyzed samples. Results are expressed as the mean ± SD (n = 3).

**Table 3:** Enzymatic activity of polyphenol oxidase (PPO), pectin methylesterase (PME), and peroxidase (POD) of *Opuntia ficus-indica* cv ‘Rossa’ juice and fermented beverages.

Sample	PPO (∆Abs_420_ nm/min/mL)	PME (V_NaOH_/min/mL)	POD (∆Abs_414_ nm/min/mL)
Juice	0.679 ± 0.026 ^a^	0.477	0.195
F18	0.585 ± 0.002 ^b^	0.134	0.02
F18-HPP	0.497 ± 0.028 ^c^	0.0855	0.015
F18-TP	0.417 ± 0.003 ^d^	0.0316	0.015
F42	0.351 ± 0.031 ^e^	0.0631	0.003
F42-HPP	0.247 ± 0.032 ^f^	ND	0.003
F42-TP	0.266 ± 0.017 ^f^	ND	0.005

F18 and F42- beverages obtained by fermentation for 18 h and 42 h, respectively, without pasteurization procedure; F18-HPP and F42-HPP- beverages obtained by fermentation for 18 h and 42 h, respectively, and post-fermentation pasteurization by high-pressure; F18-TP and F42-TP- beverages obtained by fermentation for 18 h and 42 h, respectively, and post-fermentation pasteurization by thermal pasteurization. ND: non-detected. Data marked with different letters are significantly different (ANOVA test; *p* ≤ 0.01) between treatments (in column) of the analyzed samples. Results are expressed as the mean ± SD (n = 3).

**Table 4:** Phytochemical content given by the total phenolic content (TPC), total flavonoid content (TFC), and betalain content of *Opuntia ficus-indica* cv ‘Rossa’ juice and fermented beverages.

Sample	TPC mg_GAE_/mL	TFC mg_QEq._/L	Betalain		
			Betacyanin mg_Betacyanin_/L	Betaxanthin mg_Betaxanthin_/L	Total Betalain mg_Tbetalain_/L
Juice	0.637 ± 0.133 ^a^	8.12 ± 0.1 ^a^	28.210 ± 0.085 ^a^	24.890 ± 0.085 ^a^	53.100
F18	0.613 ± 0.032 ^a^	7.62 ± 0.3 ^a^	26.332 ± 0.351 ^a^	22.244 ± 0.405 ^b^	47.430
F18-HPP	0.545 ± 0.149 ^a^	6.41 ± 0.4 ^a^	27.165 ± 0.430 ^a^	24.436 ± 0.381 ^a^	52.174
F18-TP	0.487 ± 0.103 ^a^	5.39 ± 0.5 ^a^	25.243 ± 0.104 ^b^	21.764 ± 0.253 ^b^	45.807
F42	0.473 ± 0.129 ^a^	6.21 ± 0.3 ^a^	23.677 ± 0.519 ^b^	19.381 ± 0.484 ^b^	42.059
F42-HPP	0.445 ± 0.042 ^b^	5.33 ± 0.5 ^a^	21.954 ± 0.480 ^b^	21.367 ± 0.504 ^b^	43.321
F42-TP	0.430 ± 0.005 ^b^	7.64 ± 0.6 ^a^	20.405 ± 0.053 ^b^	21.193 ± 0.123 ^b^	41.598

F18 and F42- beverages obtained by fermentation for 18 h and 42 h, respectively, without pasteurization procedure; F18-HPP and F42-HPP- beverages obtained by fermentation for 18 h and 42 h, respectively, and post-fermentation pasteurization by high-pressure; F18-TP and F42-TP- beverages obtained by fermentation for 18 h and 42 h, respectively, and post-fermentation pasteurization by thermal pasteurization. Data marked with different letters are significantly different (ANOVA test; *p* ≤ 0.01) between treatments (in column) of the analyzed samples. Results are expressed as the mean ± SD (n = 3).

**Table 5:** Identification of the phytochemicals detected in the chromatograms of *Opuntia ficus-indica* cv ‘Rossa’ juice and fermented beverages before and after pasteurization, at 280 nm, by UHPLC-DAD-ESI-MS^n^.

Peak	RT (Min)	UV (λ_max_)	[M-H]- (m/z)	MS/MS (m/z)	Identification
1	1.4	245	191	209	Quinic acid
2	1.5	204, 242	133	115, 133	Malic acid
3	1.7	210,325	179	89	Caffeic acid
4	1.8	206, 253	191	111, 173	Citric acid
5	2.3	270, 534	269	179	Not identified
6	3.7	224, 274	255	165, 193	Piscidic acid
7	6.3	219, 278	253	177	Not identified
8	8.6	224, 272	239	179, 149	Eucomic acid
9	10.8	229, 270	613	405	Syrinigil (t8-O-4) guaiacyl
10	11.1	254, 352	769	315, 338	Isorhamnetin *O*-hexoxyl-di-deoxyhexoside
11	11.5	254, 352	755	315	Isorhamnetin *O*-pentosyl-rutinoside
12	12.1	251, 351	609	315, 314	Isorhamnetin *O*-pentosyl-hexoside
13	12.7	254, 345	623	315	Isorhamnetin *O*-rutinoside

**Table 6:** Antioxidant activity of *Opuntia ficus-indica* cv ‘Rossa’ juice and fermented beverages.

Samples	ABTS^•+^ (mg_GAE_/mL)	DPPH^•^ (mg_GAE_/mL)	NO^•^ (mg_GAE_/mL)	SO^•^ (mg_GAE_/mL)	Reducing Power (mg_BHAE_/mL)
Juice	0.093 ± 0.001 ^a^	0.069 ± 0.004 ^a^	0.084 ± 0.006 ^a^	1.00 ± 0.001 ^a^	0.676 ± 0.066 ^a^
F18	0.065 ± 0.004 ^b^	0.070 ± 0.018 ^a^	0.120 ± 0.001 ^b^	1.07 ± 0.006 ^b^	0.661 ± 0.065 ^a^
F18-HPP	0.084 ± 0.003 ^ca^	0.119 ± 0.045 ^b^	0.115 ± 0.014 ^b^	1.03 ± 0.015 ^c^	0.681 ± 0.002 ^a^
F18-TP	0.079 ± 0.010 ^cab^	0.069 ± 0.015 ^ca^	0.086 ± 0.010 ^ba^	1.10 ± 0.009 ^d^	0.521 ± 0.017 ^b^
F42	0.066 ± 0.001 ^cb^	0.066 ± 0.010 ^ca^	0.100 ± 0.012 ^ba^	1.11 ± 0.005 ^d^	0.686 ± 0.018 ^a^
F42-HPP	0.075 ± 0.003 ^cb^	0.060 ± 0.006 ^ca^	0.084 ± 0.004 ^ba^	1.08 ± 0.008 ^eb^	0.563 ± 0.099 ^ab^
F42-TP	0.078 ± 0.003 ^cab^	0.059 ± 0.008 ^ca^	0.078 ± 0.004 ^ca^	1.08 ± 0.009 ^ebd^	0.522 ± 0.058 ^b^

F18 and F42- beverages obtained by fermentation for 18 h and 42 h, respectively, without pasteurization procedure; F18-HPP and F42-HPP- beverages obtained by fermentation for 18 h and 42 h, respectively, and post-fermentation pasteurization by high-pressure; F18-TP and F42-TP- beverages obtained by fermentation for 18 h and 42 h, respectively, and post-fermentation pasteurization by thermal pasteurization. Data marked with different letters are significantly different (ANOVA test; *p* ≤ 0.01) between treatments (in column) of the analyzed samples. Results are expressed as the mean ± SD (n = 3).

## Data Availability

The data that support the findings of this study are available within the article.
